# Cervical squamous cell carcinoma with isolated tibial metastasis: A case report and review of the literature

**DOI:** 10.3892/ol.2014.2540

**Published:** 2014-09-16

**Authors:** FANG YUAN, CHUNMEI ZHANG, ZHUMEI CUI, XIANG LI, XIA LI, WEI LIN, XINGSHENG YANG

**Affiliations:** 1 Department of Obstetrics and Gynecology, Qilu Hospital, Shandong University, Jinan, Shandong 250012, P.R. China; 2 Department of Gynecology, The Affiliated Hospital of Qingdao University, Qingdao, Shandong 266000, P.R. China; 3 Reproductive Medicine Center, Peking University Third Hospital, Beijing 100000, P.R. China; 4 Department of Pathology, The Affiliated Hospital of Qingdao University, Qingdao, Shandong 266000, P.R. China

**Keywords:** cervical carcinoma, bone metastasis, tibia

## Abstract

Bone metastasis resulting from carcinoma of the cervix is rare, particularly in the isolated distal appendicular bone. A 43-year-old female was diagnosed with a right tibial tumor with progressive right knee pain for three months, which was diagnosed as poorly differentiated metastatic squamous cell carcinoma, and further confirmed by biopsy of the proximal tibia. The patient was diagnosed with cervical squamous cell carcinoma with tibial metastasis following further examination, despite a lack of gynecological symptoms. In contrast to the poor outcome commonly observed in patients with bone metastasis, the patient survived and remained disease-free 41 months after surgical excision of the metastatic tumor and radical hysterectomy followed by chemoradiotherapy. The present case is one of the few documented cases of metastasis to the tibia arising from carcinoma of the uterine cervix and may be the first regarding isolated metastasis at this site.

## Introduction

As the second most common type of cancer in females worldwide, cervical cancer is characterized by a range of minor to severe neoplastic changes in the epithelium. More than 80% of the estimated 530,000 novel cases and 275,000 mortalities due to cervical cancer each year occur in the developing countries (1). The most common symptoms of cervical cancer are unusual vaginal bleeding and persistent vaginal discharge that is blood-stained or smells unpleasant. Unusual vaginal bleeding is more likely to be present at earlier stages of the disease (2). Cervical cancer diagnoses made at an earlier stage according to International Federation of Gynecology and Obstetrics (FIGO) (stages 1a1 to 1b2) are associated with higher survival rates (80–99%) compared with diagnoses made at a later stage (stages III–IV) which have associated five-year survival rates of 20–50%) (2).

An abdominal radical hysterectomy (RAH) with pelvic lymphadenectomy is the standard treatment for cervical cancer patients at stages Ia2-IIa. For patients who develop locally advanced cervical cancer, the standard of care has evolved from external beam radiation therapy (EBRT) alone, to EBRT plus brachytherapy, to combined EBRT plus brachytherapy with concurrent chemotherapy (3)

Cervical carcinoma metastasizes predominantly via direct extension and the lymph nodes, while the hematogenous route is relatively rare. Lung and supraclavicular lymph nodes are common sites for distant invasion. Lung and supraclavicular lymph nodes are common sites for distant invasion. Bone metastasis is rare, predominantly involving the vertebrae, followed by the pelvic bone, while uncommon in the distal appendicular bone (4,5). Bone metastasis is indicative of a poor prognosis and patient quality of life is severely affected. The present study reports a case of isolated tibial metastases secondary to cervical carcinoma, with a review of the relevant literature. Written informed consent was obtained from the patient.

## Case report

### Clinical presentation

**Figure 1:**
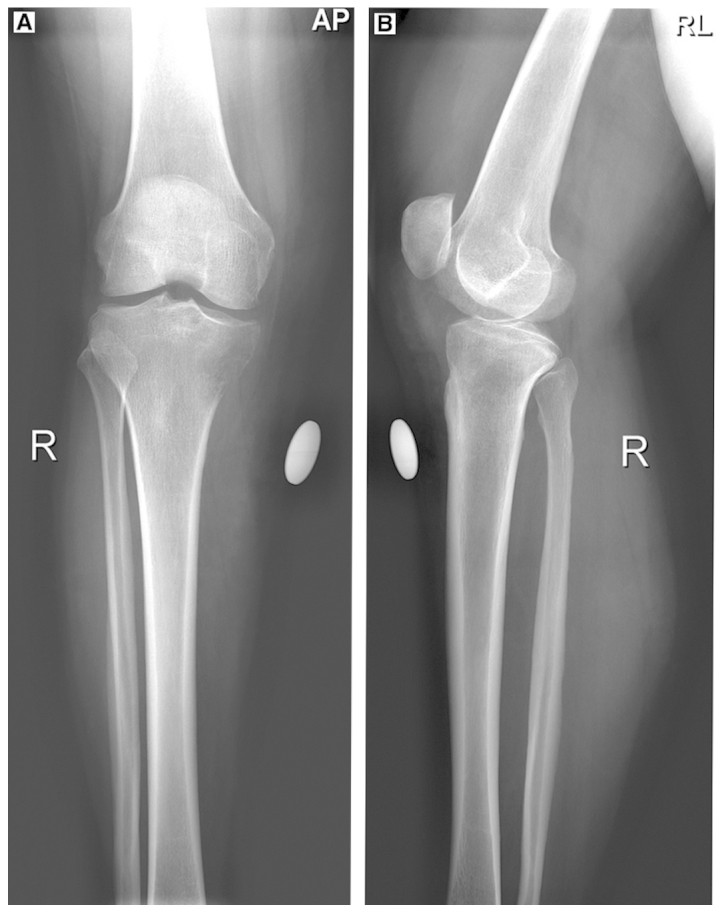
X-ray scan revealed the presence of a space-occupying lesion in the right knee medial tibia platform. (A) Posteroanterior examination and (B) lateral examination.

On February 20th 2010, a 43-year-old female was admitted to the Affiliated Hospital of Qingdao University (Qingdao, China), with progressive right knee pain of three months. Physical examination revealed a marginally swollen right knee with medial tenderness and limited flexion activity. X-ray indicated the presence of a space-occupying lesion in the right knee medial tibia platform (Fig. 1). Subsequently, the patient was diagnosed with a right tibial tumor.

**Figure 2:**
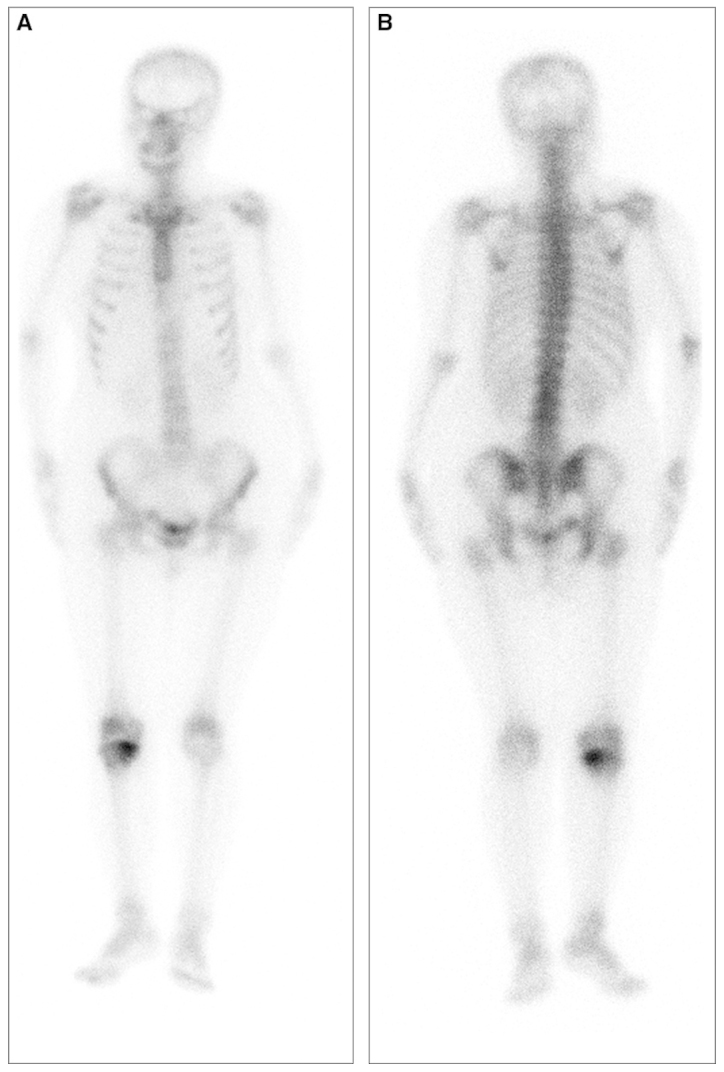
Emission computed tomography revealed an abnormal concentration of radioactive material in the inner side of the upper tibiae. The bones of the rest of the body were normal. (A) Anterior surface and (B) posterior surface.

**Figure 3:**
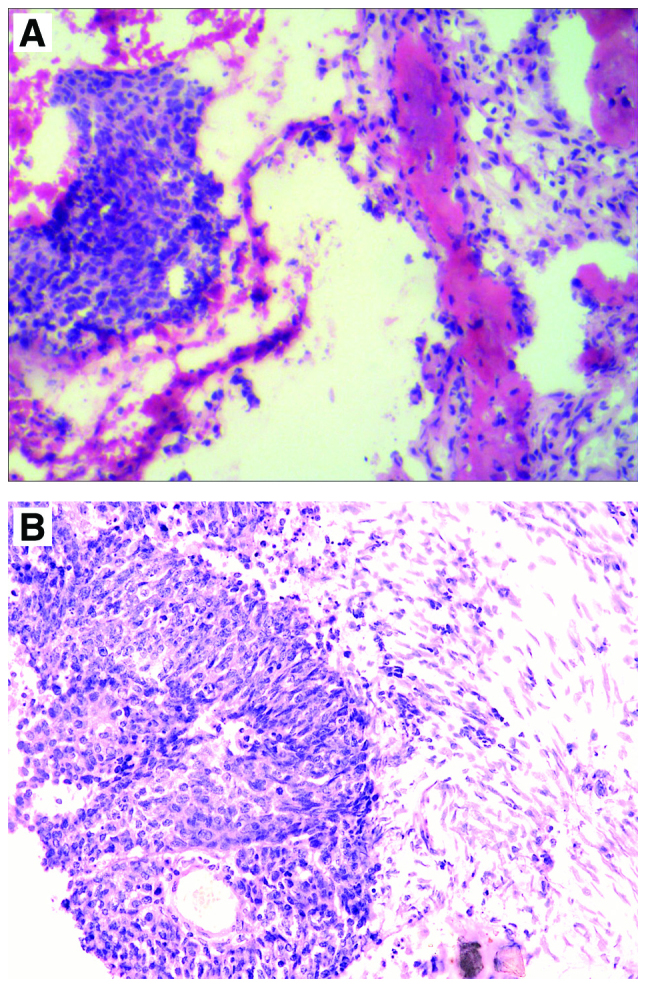
(A) Poorly differentiated metastatic squamous cell carcinoma of the tibia. (B) Poorly differentiated cervical squamous carcinoma. Stain, hematoxylin and eosin; magnification, ×200.

Emission computed tomography (ECT) demonstrated an abnormal concentration of radioactive material in the inner side of the upper tibia. The bones of the rest of the body were normal (Fig. 2). Biopsy of proximal tibiae revealed the presence of poorly differentiated metastatic squamous cell carcinoma (Fig. 3). The patient underwent right tibia tumor resection and an allogeneic bone graft.

Further examination by a gynecologist revealed that the patient exhibited prolonged menstruation accompanied by subclinical vaginal discharge for three months. Gynecological examination identified a mildly erosive cervix, with a marginally hypertrophic anterior lip and the uterine body was at a size comparable to that observed in females who are ~40 days pregnant; however, no adnexal masses were identified. Color Doppler ultrasound revealed a highly vascularized hypo echoic lesion extending from the lower segment of the uterine body to the cervix (5.3×3.3×4.0 cm) with an irregular shape and unclear boundary from the endometrium. Computed tomography (CT) revealed an enlarged uterine body lesion, occupying the left inguinal area and multiple hyper-density masses in the mediastinum. Liquid-based cervical cytology indicated negativity for intraepithelial lesions or malignancy. The levels of tumor markers were all within normal ranges; serum cancer antigen 125 (CA125), CA199 and carcinoembryonic antigen levels were 19.15 kU/l, 2.57 kU/l and 0.73 μg/l, respectively. The pathology of fractional curettage and cervical biopsy revealed poorly differentiated squamous cell carcinoma in the cervical canal and lower segment of the uterine cavity. The patient was diagnosed with FIGO stage Ib2 cervical squamous cell carcinoma with tibial metastasis (6).

### Treatment and outcome

The patient underwent extensive hysterectomy, bilateral adnexectomy, and pelvic and para-aortic lymph node dissection. Intra-operative examination revealed that the size of the uterus was comparable to that of a female who is ~40 days pregnant, and the lower segment was enlarged. The right common and external iliac lymph nodes, as well as the para-aortic lymph nodes, were enlarged and hard. Interventional chemotherapy with 60 mg cisplatin and 500 mg fluorouracil was administered via the bilateral internal iliac arteries. Postoperative histopathological examination revealed poorly differentiated cervical squamous cell carcinoma with para-aortic lymph node metastasis (Fig. 3) and endometrial complex hyperplasia, with regional moderately atypical hyperplasia. The patient underwent six courses of chemotherapy with cisplatin and paclitaxel, as well as pelvic radiotherapy. Following chemotherapy, the patient was evaluated by pelvic and thoracic CT, as well as tibia and fibula X-rays. The results of the additional examinations were normal. During the follow-up period of 41 months, no metastasis or recurrence was identified.

## Discussion

Cervical carcinoma is one of the most common types of malignant tumor in females. The incidence of bone metastasis is clinically confirmed as ~1.1–8.2% (7–10). However, based on autopsy data, bone metastasis incidence is as high as 8.6–17.9%, which is significantly higher than that identified clinically (11,12). In a study by Yoon *et al* (13), 105 patients with bone metastasis with invasive carcinoma of the uterine cervix were retrospectively analyzed. The authors demonstrated that adenocarcinoma, advanced stage (IIB–IV) and initial multiple bone metastases contribute to earlier bone metastasis. Cervical squamous cell carcinomas metastasize predominantly via direct extension and the lymph nodes, while the hematogenous route is relatively rare. Bone metastasis is rare, predominantly involving the vertebrae, followed by the pelvic bone, while uncommon in the distal appendicular bone (4,5). A number of studies have reported cases of isolated localized metastasis to the fibula, patella and humerus, arising from cancer of the uterine cervix (14–16). To the best of our knowledge, the present case of isolated tibial metastasis arising from cervical squamous cell carcinoma is even rarer. In the present case, the patient exhibited no evidence of a clinical gynecological disorder until the tibial tumor was diagnosed.

In clinical practice, bone metastases arising from malignant tumors usually present as severe and progressive pain in metastatic sites, and pathological fracture may occur (10). X-ray, total bone ECT examination, needle biopsy of the local lesion and positron emission tomography (PET)-CT scans are useful methods used to detect lesions of advanced cervical carcinoma (17). The diagnostic criteria of bone metastasis are as follows (18): (i) Local intermittent or persistent bone pain; (ii) total body bone ECT examination showing abnormal radioactive material accumulation at the site of the lesion, the concentration of which is higher than that of the contralateral or adjacent areas; (iii) X-ray, CT or magnetic resonance imaging examination demonstrating the presence of osteolytic or osteoblastic changes, bone uplift and pathological fractures; and (iv) follow-up confirming persistent existence or further extensions of the lesion. Diagnosis is established if the criteria of (i), (ii) and (iv) or (i), (iii) and (iv) are met. Biopsy is the ‘gold standard’ for the detection of bone metastasis arising from cervical carcinoma (19). Clinical manifestations, combined with corresponding imaging examination such as X-ray, CT or MRI and histopathology contribute to the early diagnosis of bone metastasis arising from cervical carcinoma. The predominant symptom exhibited in the present case was progressively aggravated pain in the right knee. ECT examination revealed radioactive material accumulation in the proximal end of the right tibia, and biopsy confirmed the pathology of poorly differentiated squamous cells. Finally, the detection of a lesion exhibiting the same phenotype in the cervix further confirmed the diagnosis.

Malignant tumors with bone metastases require standardized guidelines regarding the therapeutic options. Current treatments focus on pain relief, prolonging patient life and preservation of patient quality of life (15). Previous studies have shown that short-cycle radiotherapy can be as effective as long-cycle radiotherapy in alleviating pain caused by bone metastases (20). Generally, surgical management or fixation of pathological fracture is recommended in cases of good ECOG performance status (21) with solitary bone metastasis, supplemented by palliative radiotherapy (22). Pasricha *et al* (14) indicated that surgery combined with concurrent chemoradiotherapy may achieve better effects for patients with metastases who are in good physical condition. Diphosphonates used in palliative treatment have been demonstrated to be effective in relieving pain, reducing the occurrence of pathological fractures and improving quality of life; however, they did not prolong survival time (23). Another study found that PET-CT-guided treatments improved the overall survival rates of patients with locally advanced cervical cancer (24). The patient in the present case underwent aggressive treatment of the primary tumor, supplemented with chemotherapy. A positive response to treatment was observed and the patient was free of disease at the 41-month follow-up visit.

Pasricha *et al* (14) presented a case of FIGO stage IIB cervical carcinoma. The patient developed fibula metastasis nine months following radiotherapy and underwent surgical excision of metastatic lesion; however, the patient refused further salvage chemotherapy. The patient succumbed to the disease 48 months following the presentation of primary symptoms. Furthermore, Corrado *et al* (15) presented a patient with poorly differentiated cervical adenosquamous carcinoma in stage IIB who underwent surgical resection of a femoral metastatic lesion. After three months the patient succumbed to the disease as concurrent chemoradiotherapy was refused. It has been reported that bone metastasis usually occurs within two years of the diagnosis of the primary tumor. Patients generally succumb to the disease within 18 months of the diagnosis of bone metastasis, which indicates a poor prognosis (22). Abdul-Karim *et al* (11) analyzed 20 cases of cervical carcinoma with bone metastases and found that 71% of bone metastases occurred within two years of diagnosis. Zhao *et al* (24) performed a clinical analysis of eight patients with bone metastases from uterine malignant tumor, and found that occurrence times were similar to those reported in a study by Abdul-Karim *et al* (11). Previous studies have shown that the prognosis of patients with bone metastasis was associated with metastatic site (isolated or multiple bone metastases), and whether lymph nodes or other organs were involved (24). Other factors, including whether treatments of primary malignant tumor were standardized or therapeutic options for bone metastases such as surgery, EBRT, EBRT plus brachytherapy, or combination EBRT plus brachytherapy with concurrent chemotherapy, have been found to contribute to the prognosis (24).

The primary symptom presented by the patient in the current case was local pain in the metastatic bone without evident manifestation of the primary tumor. Therefore, a detailed investigation of medical history and comprehensive physical examination are required when clinicians diagnose patients with local bone pain, in order to avoid any delay in treatment. The patient underwent surgery (RAH with a pelvic lymphadenectomy) for the primary tumor when a definitive diagnosis had been made, which was then followed by chemoradiotherapy. The patient responded well to the treatments, and was free of recurrence and metastasis at the 41-month follow-up visit. Therefore, this case indicated that aggressive and acurate treatments are beneficial for advanced cancer patients with bone metastases. A combination of standardized surgical treatment and chemoradiotherapy must be recommended to achieve the best outcome and improve patient quality of life.l

## References

[1] Pan QJ, Hu SY, Guo HQ (2014). Liquid-based cytology and human papillomavirus testing: a pooled analysis using the data from 13 population-based cervical cancer screening studies from China. Gynecol Oncol.

[2] Low EL, Simon AE, Lyons J (2012). What do British women know about cervical cancer symptoms and risk factors?. Eur J Cancer.

[3] Banerjee R, Kamrava M (2014). Brachytherapy in the treatment of cervical cancer: a review. Int J Womens Health.

[4] Friedlander M, Grogan M, U.S. Preventative Services Task Force (2002). Guidelines for the treatment of recurrent and metastatic cervical cancer. Oncologist.

[5] Kanayama T, Mabuchi S, Fujita M, Kimura T (2012). Calcaneal metastasis in uterine cervical cancer: a case report and a review of the literature. Eur J Gynaecol Oncol.

[6] Pecorelli S (2009). Revised FIGO staging for carcinoma of the vulva, cervix, and endometrium. Int J Gynaecol Obstet.

[7] Barmeir E, Langer O, Levy JI, Nissenbaum M, DeMoor NG, Blumenthal NJ (1985). Unusual skeletal metastases in carcinoma of the cervix. Gynecol Oncol.

[8] Matsuyama T, Tsukamoto N, Imachi M, Nakano H (1989). Bone metastasis from cervix cancer. Gynecol Oncol.

[9] Ratanatharathorn V, Powers WE, Steverson N, Han I, Ahmad K, Grimm J (1994). Bone metastasis from cervical cancer. Cancer.

[10] Thanapprapasr D, Nartthanarung A, Likittanasombut P (2010). Bone metastasis in cervical cancer patients over a 10-year period. Int J Gynecol Cancer.

[11] Abdul-Karim FW, Kida M, Wentz WB (1990). Bone metastasis from gynecologic carcinomas: a clinicopathologic study. Gynecol Oncol.

[12] Disibio G, French SW (2008). Metastatic patterns of cancers: results from a large autopsy study. Arch Pathol Lab Med.

[13] Yoon A, Choi CH, Kim HJ (2013). Contributing factors for bone metastasis in uterine cervical cancer. Int J Gynecol Cancer.

[14] Pasricha R, Tiwari A, Aggarwal T, Lal P (2006). Carcinoma of uterine cervix with isolated metastasis to fibula and its unusual behavior: report of a case and review of literature. J Cancer Res Ther.

[15] Corrado G, Santaguida S, Zannoni G, Scambia G, Ferrandina G (2010). Femur metastasis in carcinoma of the uterine cervix: a rare entity. Arch Gynecol Obstet.

[16] Malek M, Kanafi AR, Pourghorban R, Nafisi-Moghadam R (2012). Isolated humeral metastasis in uterine cervical cancer: a rare entity. J Clin Imaging Sci.

[17] Huh JW, Min JJ, Lee JH, Kim HR, Kim YJ (2012). The predictive role of sequential FDG-PET/CT in response of locally advanced rectal cancer to neoadjuvant chemoradiation. Am J Clin Oncol.

[18] Pomeranz SJ, Pretorius HT, Ramsingh PS (1994). Bone scintigraphy and multimodality imaging in bone neoplasia: strategies for imaging in the new health care climate. Semin Nucl Med.

[19] Sadik M, Suurkula M, Hoglund P, Jarund A, Edenbrandt L (2009). Improved classifications of planar whole-body bone scans using a computer-assisted diagnosis system: a multicenter, multiple-reader, multiple-case study. J Nucl Med.

[20] McQuay HJ, Carroll D, Moore RA (1997). Radiotherapy for painful bone metastases: a systematic review. Clin Oncol (R Coll Radiol).

[21] Buccheri G, Ferrigno D, Tamburini M (1996). Karnofsky and ECOG performance status scoring in lung cancer: a prospective, longitudinal study of 536 patients from a single institution. Eur J Cancer.

[22] Blythe JG, Cohen MH, Buchsbaum HJ, Latourette HB (1975). Bony metastases from carcinoma of cervix. Occurrence, diagnosis, and treatment. Cancer.

[23] Dunstan CR, Felsenberg D, Seibel MJ (2007). Therapy insight: the risks and benefits of bisphosphonates for the treatment of tumor-induced bone disease. Nat Clin Pract Oncol.

[24] Zhao Y, Wang JL, Wei LH, Bao DM (2006). Clinical analysis of eight cases of bone metastasis of uterine carcinomas. Zhonghua Fu Chan Ke Za Zhi.

